# Modular UV Curing Sol-Gel Coating for Invisible, Levelling and Easy to Clean Multi-Layer Systems

**DOI:** 10.3390/ma16155442

**Published:** 2023-08-03

**Authors:** Lucía Florentino Rico, Olga Conejero Iglesias, Ramón Bernardo de la Rua García, Jennifer Moriones Domeño, Jonathan Fernández de Ara, Gonzalo G. Fuentes

**Affiliations:** 1 Idonial Foundation, C/Calafates, 33417 Avilés, Spain; olga.conejero@idonial.com (O.C.I.); ramon.delarua@idonial.com (R.B.d.l.R.G.); 2 Surface Technologies and Advance Materials, AIN, Carretera Pamplona, 1, 31191 Cordovilla, Spain; jmoriones@ain.es (J.M.D.); jfernandez@ain.es (J.F.d.A.); gfuentes@ain.es (G.G.F.)

**Keywords:** sol-gel, UV curing, photocurable, coating, hydrophobic, easy to clean, contact angle, levelling

## Abstract

Current methods for the hardening step of functional coatings over different materials imply the use of high temperatures, high energy consumption or long periods of time, which have repercussions on the speed and cost of the product. We report here a simple and low-cost methodology for the functionalization of low-cost stainless steel, which is modular, depending on the functionality pursued: a levelling layer for smoothing the surface of the material, an “easy to clean” property, or both of them. This research is based on sol-gel coatings cured under UV light without requiring high thermal hardening processes, making it applicable to plastics and other sensible materials and possessing high chemical and thermal stability. The film ensures lower processing costs and higher rates of hardening if adequate medium-pressure lamps are employed. This formulation is also well-defined for scaling up the process, so it is possible to perform a continuous coating in large areas by employing mild processing conditions (low temperature, atmospheric pressure). In addition, the sol-gel solution was fully characterized and studied in order to guarantee a long service life before deposition, with a focus on industrial applications in the domestic sector.

## 1. Introduction

In the history of materials science, sol-gel technology has been widely used since 1980 [1,2] in the process of synthesizing superconducting materials [3], functional ceramic materials [4], nonlinear optical materials [5], catalysts and enzyme carriers for biomedical applications [6,7], porous glass and other materials [8,9]. This method used for the development of functional surfaces over multiple substrates is gaining much more attention since, for many applications, it offers a reduction in cost and complexity compared with other methodologies. Among the substrates where these coatings can currently be applied, there is a large repertoire, from fabrics for many textile applications [10] to polymers for conferring flame retardant features [11] or glass for the study of antifouling coatings for marine applications [12].

**Figure 1:**
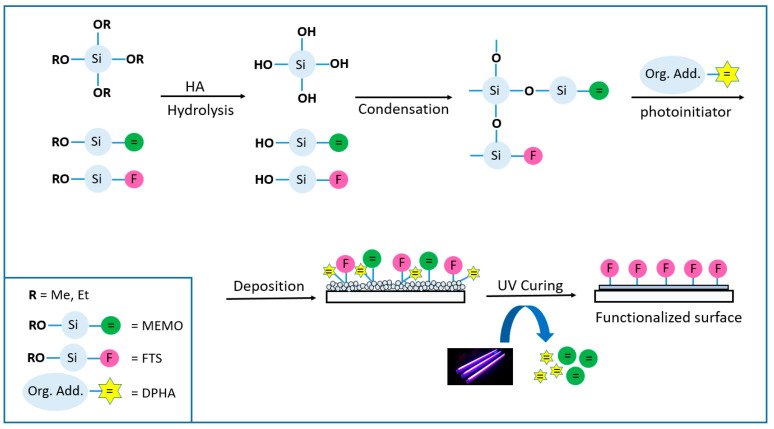
Photocurable sol-gel reaction scheme. Coating procedure.

Sol-gel SiO_2_ coatings are obtained mainly by dissolving an alkoxide precursor in alcohol together with the appropriate additives, which are then hydrolyzed in water in the presence of an acid or basic catalyst source. Once the hydrosilylation reaction takes place, the alcohol molecules are condensed, forming a cross-linked polysiloxane network, and at this point, the gelation process starts. After the deposition process of the solution, prepared by spinning, dipping or spraying (among others) over the substrate, it is necessary to carry out densification of the layer to complete the gelification process by heating at high temperatures or by polymerization under UV light (technology considered in this work), leading to a compact layer free of cracks or striations, chemically and physically resistant (Figure 1).

Research on sol-gel coatings cured under UV light without requiring high-temperature hardening processes on plastic surfaces has recently been the center of attention. In addition to the low-temperature curing process, the UV-cured films have various advantages, such as high chemical and thermal stability, less harm to the environment, lower processing costs, and higher rates of hardening if the appropriate medium-pressure lamps are employed.

A photopolymerisable coating formulation essentially consists of adding a polymerizable molecule (oligomer, in the present work, dipentaerythritol penta-/hexa-acrylate_DPHA) and a light-sensitive compound or photoinitiator that is able to convert the absorbed light energy into a more useful form capable of causing the binder to polymerize into a hard solid film. The photoinitiator produces initiator radicals directly by the fragmentation of the photo-excited state, and for this kind of coating, aromatic α-hydroxiketones such as 1-hydroxycyclohexyl phenyl ketone (Irgacure 184) or 2-Hydroxy-2-methylpropiophenone (Darocure 1173) were suitable candidates [13]. In these systems, photoinitiators mainly affect cure speed, yellowing, and cost. In addition, the light absorption properties of the selected photoinitiator need to match the emission wavelength of the light source [14]. Figure 1 represents the general process for this technology, where it is noticeable how the double bonds of the acrylate molecules are eliminated upon radical reactions and co-polymerization of the different chemicals involved in the process (3-(Trimethoxysilyl) propyl methacrylate_MEMO and the acrylic monomer DPHA) causing the total gelification of the coating.

Organic-inorganic hybrid sol-gel coatings (OIH) were studied for their application over stainless steel [15] and other metal substrates [16,17,18] in order to evaluate corrosion protection [19]. Intermolecular interactions between the macromolecular existing structures and the metallic surface are reviewed in the literature to achieve low porosity, rigidity and adhesion to the metallic substrate, creating a good physical barrier against oxidation, corrosion and erosion, among other processes.

A model of protective coating produced in five steps using mainly sol-gel methods was studied by Hughes et al. in the form of a multilayer coating system with a total thickness of about 16 µm [20]. They propose a protection with dual behavior: a protective barrier taking advantage of the OIH properties with a self-healing response. In this multilayer system, the formulation of the different layers is based on organic-inorganic hybrid sol-gel matrix composition because of the advantage of being produced at room temperature conditions on an industrial scale by using well-established and low-cost methods.

The first aim of this work is to analyze the levelling capability of a sol-gel coating onto low-cost metallic substrates to prepare the material for an additional decorative layer deposited by Physical Vapor Deposition, which requires a smooth surface to adhere. Although there are many studies in the literature based on levelling thermally cured sol-gel coatings, to our knowledge, the UV curing version for this application has not been studied.

For example, Guillen et al. studied the levelling capability of sol-gel SiO_2_ layers onto titanium and Kovar (Fe54 Ni29 Co17) foil substrates [21]. In this work, the coatings were prepared by immersion techniques and the roughness of the coating was determined for different withdrawal rates, and therefore the thickness of the film after the dip-coating deposition method. The sintering step of the sol-gel layers was obtained by heat treating in the air at 400 °C for 1 h, and the Ra levelling parameter was improved (decreased) by adding successive sol-gel SiO_2_ layers with their corresponding heating treatment.

Similarly, a method for the functionalization of low-cost steel with application on solar cells was recently described, with the properties of surface levelling, electrical insulation and anti-diffusion barrier [22]. The application method for this work was spray coating, and the samples were cured by the near-infrared technique (NIR) at an irradiation power of 90%. A curing time of 2 min was applied in all cases (reaching a peak metal temperature of 198 °C), which represents a clear time reduction compared to conventional hot-plate curing (typically close to one hour).

In a thermal curing process, densification of the layer is usually obtained by heating at temperatures over 400 °C. By using the photocurable method, the levelling layer could be performed under mild processing conditions (low temperature and atmospheric pressure). For this purpose, AISI 430 stainless steel foils were covered with the optimal formulation parameters at various thicknesses, first by spin coating and later by spraying techniques, in a large-scale study. The analysis of the roughness, topography and morphology of these samples allowed us to conclude that a levelling functionality of the film has been achieved, with good adhesion properties and employing ultrafast curing methods.

On the other hand, we studied the addition of a fluoro-compound with a low surface energy in the appropriate step of the formulation to convert the previously designed coating onto a hydrophobic surface, thereby easily achieving a new functionality for different materials just by adding an extra additive. There are few examples in the literature for this kind of UV-curable coating [23,24], but all of them are focused exclusively on the coating of plastics such as polymethyl methacrylate or polycarbonate, and not applicable to metallic surfaces, requiring an extra step of heating before UV curing and applied by different methods in small samples.

Here we report the optimized formula for a dual-functional coating onto stainless steel surfaces, not only for laboratory scale but also for covering large areas and different geometries by employing different deposition methods.

## 2. Materials and Methods

### 2.1. Materials and Chemicals

An AISI 430 stainless steel sheet with a thickness of 0.3 mm was cut into 50 × 50 mm^2^ or 100 × 100 mm^2^ sized substrates, depending on the scale-up of the formulation and the application method studied. After cleaning with acetone, detergent and surfactant ultrasonically for 15 min, the samples were rinsed with distilled water and anhydrous ethanol and then dried with air. The AISI 430 chemical composition, analyzed by arc spark spectroscopy, is reported in Table 1.

Concerning the sol formulation, tetraethyl orthosilicate (TEOS) for synthesis, 3-(Trimethoxysilyl) propyl methacrylate (MEMO, 98%) and the organic crosslinking agent, dipentaerythritol penta-/hexa-acrylate (DPHA) were purchased from Sigma-Aldrich (St. Louis, MO, USA). The photo-initiator, 2-hydroxy-2-methylpropiophenone (Darocure 1173) and the fluoroalkylsilane, triethoxy-1H, 1H, 2H, 2H-tridecafluoro-n-octyltriethoxysilane (F13), were purchased from TCI (Grindelwald, Switzerland). Hydrochloric acid (37%, AnalaR NORMAPUR^®^, Reag. Ph. Eur. analytical reagent) and 2-propanol (≥99.7%, AnalaR NORMAPUR^®^, Reag. Ph. Eur. analytical reagent) were purchased from VWR (Barcelona, Spain). All materials were used as received, except for 1H, 1H, 2H, 2H-Perfluorooctyltriethoxysilane (F13), which was transferred to an opaque two-necked flask and handled under nitrogen.

### 2.2. Synthetic Procedure for the Levelling Layer

#### 2.2.1. Laboratory Scale

To prepare the sol-gel solution, 5 mL of TEOS was mixed with IPA in a molar ratio of TEOS/IPA = 2.4 to form a homogeneous solution. Then, aqueous HCl (pH 1.2) was added under agitation to induce the sol-gel reaction with an H_2_O/TEOS ratio of 4. The solution was stirred for 2 h at room temperature, after which MEMO (in a TEOS/MEMO ratio of 4.5), additional IPA (TEOS/IPA = 6.4), and additional aqueous HCl (H_2_O/MEMO = 3) were very slowly dropped into the formed silica sol. The reaction was continued for 2 h, and appropriate amounts of DPHA and photo-initiator Darocure 1173 were added to the sol under vigorous agitation in TEOS/DPHA and TEOS/1173 ratios of 7.8 and 22.6, respectively, and stirred for 45 min. Finally, the sol was filtered and uniformly spread on 50 × 50 mm^2^ AISI 430 stainless steel substrate samples by spin coating at 3000 rpm (rotation speed), followed by treatment under UVA irradiation (365 nm) with a conventional low-pressure mercury lamp with a power density of 0.2 W/cm^2^ for 15 min to yield a cured film.

#### 2.2.2. Scale Up for Continuous Coating

A re-optimization of the small-scale process parameters was necessary to achieve the levelling functionality of the coating for the scale-up process in order to obtain a homogenous coating free of cracks and ultrafast cured. In this procedure, the water and the acid amounts were recalculated and added separately but not scaled up in a linear mode. The reaction required the same amount of water to proceed, but the catalyst charge was reduced to prevent the acid from attacking the surface of the metallic substrate when added in large quantities. Additionally, the photoinitiator was slightly reduced in quantity in this procedure, taking into account the rates of the curing step and the excess of the chemical added.

To prepare the sol-gel solution, 25 mL of TEOS (25 mL) was mixed with IPA in a molar ratio of TEOS/IPA = 2.4 to form a homogeneous solution. Then, H_2_O in an H_2_O/TEOS ratio of 3.7 and concentrated HCl 37% in a TEOS/ HCl ratio of 160 were added under agitation to induce the sol-gel reaction. The solution was stirred for 2 h at room temperature, after which MEMO (in a TEOS/MEMO ratio of 4.2) and additional IPA (TEOS/IPA = 6.9) were very slowly dropped into the formed silica sol. Additional H_2_O (H_2_O/TEOS = 0.6) and concentrated HCl 37% (TEOS/ HCl = 430) were added under agitation. The reaction was continued for 2 h, and appropriate amounts of DPHA and photo-initiator, Darocure 1173, were added into the sol under vigorous agitation in TEOS/DPHA and TEOS/1173 ratios of 7.8 and 21.5, respectively, and stirred for 45 min.

Finally, the sol was filtered, diluted to a 1:3 ratio in isopropanol, reaching a volume of approximately 150 mL and a viscosity of around 8–9 cP, and uniformly spread on 100 × 100 mm^2^ AISI 430 stainless steel substrate samples using the spray method.

Spray coating was performed using a commercial air atomizing spray gun from Spraying Systems Inc., Glendale Heights, IL, USA, with a nozzle cone angle of 60°. The spray gun was mounted on an automatized spray system and moved transversely with respect to the sample movement. The 100 × 100 mm^2^ samples were coated using the following parameters: (i) a sol-gel liquid pressure of 0.3 bar, (ii) an air pressure of 0.8 bar, (iii) a holder speed of 0.9 m/min, (iv) a nozzle/sample distance of 17 cm, and (v) a one scan spray. After deposition, samples were quickly cured under UV irradiation with a medium-pressure mercury lamp Light Hammer 10 (6.59 W/cm^2^) equipped with a D-bulb located in a specific coating pilot plant installed at our organization which allows for continuous coating in large areas. By setting a conveyor belt speed of 8 m/min and a distance of 200 mm between the light source and the samples, the curing step was performed in a flash mode of 1.5 s per sample.

### 2.3. Synthetic Procedure for the Hydrofobic/Easy to Clean Coat

To prepare the sol-gel solution, 5 mL of TEOS was mixed with IPA in a molar ratio of TEOS/IPA = 2.4 to form a homogeneous solution. Then, aqueous HCl with a pH of 1.2 was added under agitation to induce the sol-gel reaction with an H_2_O/TEOS ratio of 4. The solution was stirred for 2 h at room temperature, after which MEMO (in a TEOS/MEMO ratio of 4.5), additional IPA (TEOS/IPA = 6.4), and additional aqueous HCl (H_2_O/MEMO = 3) were very slowly dropped into the formed silica sol. The reaction was continued for 2 h, and an appropriate amount of F13 was added with an F13/MEMO molar ratio of 1. The reaction was allowed to proceed for 1 h, after which DPHA and photo-initiator Darocure 1173 were added to the sol under vigorous agitation in TEOS/DPHA and TEOS/1173 ratios of 7.8 and 22.6, respectively, and stirred for 45 min. Finally, the sol was filtered and uniformly spread on 50 × 50 mm^2^ AISI 430 stainless steel substrate samples by spin coating at 3000 rpm, followed by treatment under UVA irradiation with a conventional low-pressure mercury lamp with a power density of 0.2 W/cm^2^ for 15 min to yield a cured film.

### 2.4. Characterization

The three-dimensional morphology and surface roughness of the samples were obtained using two different profilometers. A mechanical profilometer (Model XP-1, Ambios Technology, Milpitas, CA, USA, with a resolution of 5 Å, profile length of 1.25 mm and a cut-off of 0.25 mm) and an optical profilometer (WYCO-RST 500, using the vertical scanning interferometry (VSI) mode to measure the topography in an area of 0.9 × 1.5 mm). For each sample, readings were taken in at least three different positions, and the average value was taken as the final value. The magnification of the surface was analyzed with an optical microscope (DMI5000M, LEICA, Barcelona, Spain) to check for defects and surface integrity. The chemical composition of the sample surface was analyzed by an energy-dispersive X-ray spectrometer (EDS), while the chemical structure of the coating was analyzed by Fourier transform infrared spectroscopy (FTIR-ATR Nicolet 6700, Thermo Fisher Scientific, Waltham, MA, USA). The FTIR spectrum was obtained in the infrared region from 650 to 4000 cm^−1^ using a horizontal attenuated total reflectance device. All the spectra were recorded at 2 cm^−1^ resolution. Reflectance spectra were performed for the hybrid coating after UV curing. Thermogravimetric analysis (TGA) of the sol-gel solution and UV-cured coatings was performed by using a TGA/DSC Mettler-Toledo thermogravimetric analyzer. The tests were run from 20 to 800 °C with a heating rate of 10 °C/min under an air atmosphere for the solution and under nitrogen for the UV-cured films. The static water contact angle was measured by a contact angle instrument (OCA 25, DataPhysics, Filderstadt, Germany) using a droplet size of 1 µL. The contact angles were obtained by averaging the measured values at four different locations on the inspected surface. The bonding strength between the coating and substrate was measured by cross-hatch testing in accordance with ISO 2409:2020 standard [25]. The hardness of the coatings was measured by nanoindentation employing a NanoIndenter XP (MTS) with a Berkovich diamond tip. The tip was calibrated on a fused silica sample using the Oliver and Pharr method [26]. The viscosity of the solutions was monitored using a Brookfield DVI-Prime viscosimeter. Physical vapor deposition (PVD) coatings were deposited in commercial equipment (xPro4C PVT, Bensheim, Germany) equipped with four sputtering targets (700 × 105 mm). CrN and TiN coatings were deposited using the HIPIMS technique at a controlled temperature of 80 °C.

## 3. Results and Discussion

### 3.1. Levelling Layer

**Figure 2:**
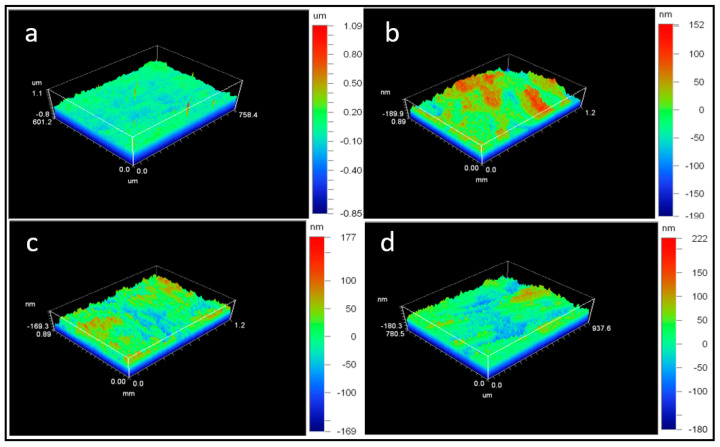
Three-dimensional images obtained through optical profilometry of the surface of the studied samples: (**a**) AISI 430 SS; (**b**) Sample A; (**c**) Sample B; (**d**) Sample C.

The topography of the AISI 430 stainless steel substrate was studied before coating, and a map obtained by optical profilometry is shown in Figure 2a. The average surface roughness (Ra) and the peak-to-valley roughness (Rt) of the uncovered substrate were determined by taking five different measurements along the surface, and the mean values were found to be 81 nm and 1.80 µm, respectively.

Different samples (as shown in Table 2) were coated via spin coating at various rotation speeds (rpm) in order to change the thickness of the layer. In general, the higher the velocity of the spin coater, the thinner the layer (as can be proved by the decrease in the thickness of the first layer, detailed in Table 2). The thickness was measured employing a mechanical profilometer. Additionally, the deposition of two consecutive layers was studied to analyze the effect on functionalization and to enhance the thickness in a different way by depositing a layer, UV curing, depositing the top coat and UV curing again. The average surface roughness (Ra) and the peak-to-valley roughness (Rt) of these samples were also measured at five different points per sample (as shown in Figure 2b–d) to quantify the levelling functionality of the sol-gel coating and the influence of the thickness on the levelling effect.

**Figure 3:**
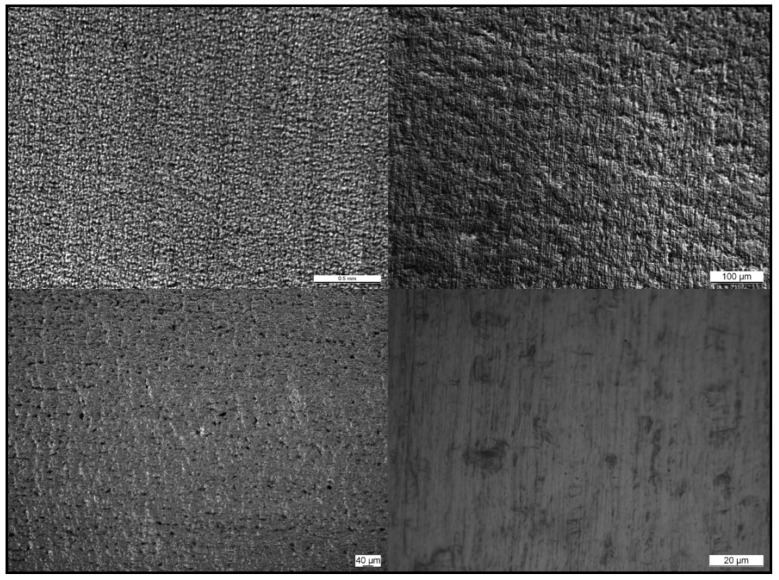
Magnification of the surface of AISI 430 SS covered with a 1.7 µm-thick SiO_2_ layer to the microscale.

The deposition of the sol-gel coating on AISI 430 stainless was designed to act as a levelling layer, independent of the thickness of the coating and the number of layers deposited (as shown in entries 2–5 in Table 2). In fact, the average surface roughness (Ra) of these samples was reduced to one-quarter of their value in the uncoated substrate, and the peak to valley roughness (Rt) decreased to around 200 nm compared to the uncoated substrate, where this value was 1800 nm (as shown in entry 1, Table 2). It was also shown that it was possible to achieve various thicknesses of the coating by slowing down the spinning of the coater or by introducing multiple layers on top, regardless of maintaining the levelling functionality intact. The surface did not present visible defects, and uniformity was observed when sample D (entry 5, Table 2) was analyzed with an optical microscope (as shown in Figure 3).

- Adhesion test

**Figure 4:**
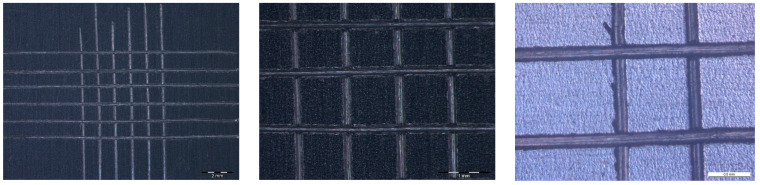
Magnification of the surface and cuts of AISI 430 SS covered with a 1.7 µm-thick SiO_2_ layer after the adherence test performed.

Good adhesion to the substrate and mechanical properties of the coating are critical points to meet the surface properties required [27]. In order to guarantee the adherence and permanence of the coating, an adhesion test (cross-cut test) was performed on a sample coated with the sol-gel studied in accordance with ISO 2409:2020 standard. The test involved making six vertical and six horizontal cuts, forming squares of 1 mm × 1 mm. An adhesive tape was applied and then removed from the cut area of the coated sample. Some pictures of the tested area were taken with an optical microscope (as shown in Figure 4), which showed that the substrate AISI 430 was exposed in the area where the coating was removed. It was observed that the coating pulled out only in the region of the incision without causing damage to the rest of the film. This result indicates that the sol-gel layer exhibited strong adherence to the substrate, which is an essential factor in guaranteeing a long service life of the final product.

- Hardness

In these types of coatings, hardness is usually quantified as pencil hardness since they are more flexible and soft coatings than thermally cured ones. Although only planar samples were coated in this study, these features allow the coating of many different geometries without disrupting the layer integrity. Nevertheless, a pencil hardness of 6H is considered hard. The hardness of the cured films in this work was examined by the pencil test, giving a result of 9H, which is the maximum value at which the coating could not be torn off the substrate. Hardness was also examined by nanoindentation [28] in order to determine the coating’s mechanical characteristics and to compare it with other coatings in the literature. In this approach, a defined indenter tip is forced into a specified spot in the test sample, and gradual unloading is conducted until the required depth is achieved. The analysis was performed in four equivalent samples with similar coating thickness, and two different forces of 500 and 1000 µN were applied (higher forces were too deep in the coating for a reliable measurement). When 1000 µN were applied, the values of hardness and Young modulus were slightly higher, indicating that the proximity of the substrate was influencing the measurement at some level. The results are gathered in Table 3, showing good repeatability, which translates to good homogeneity of the layer. The values of hardness around 0.3 GPa indicate the typical low hardness of these films, and the elastic modulus showed quite good elasticity when compared with coatings cured under thermal conditions.

**Figure 5:**
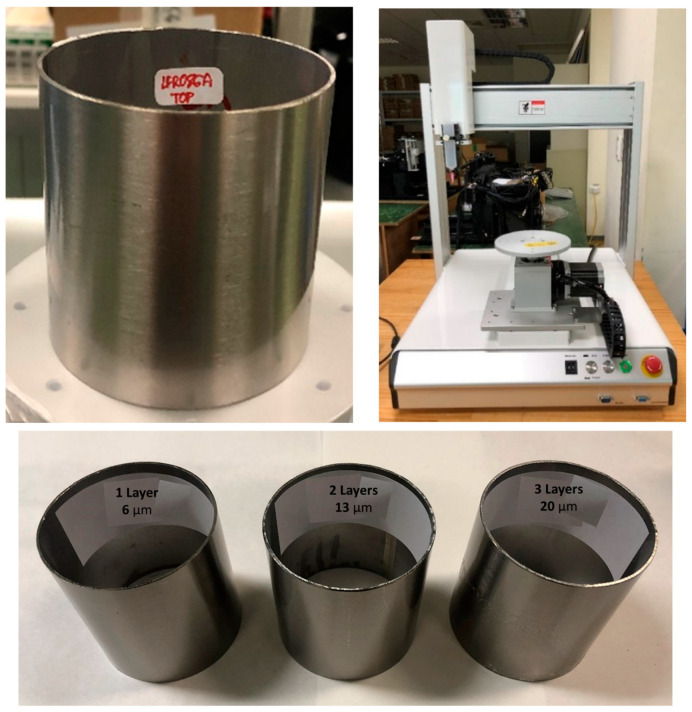
Stainless steel cylinder coated with the sol-gel formulation using a robotic spray coating system and thickness of the coating after deposition of multiple layers.

To demonstrate the feasibility of the method for coating different geometries, thanks to the good elasticity of the coating, a stainless steel cylinder was coated using the spray coating technique (as shown in Figure 5) after following the procedure described in Section 2.2.2. For this purpose, an automated robotic spray coating system (Fisnar F4400N) was employed. The best conditions were studied to achieve a uniform and continuous coating with a thickness of around 6 µm over the entire surface. The following parameters, after diluting the solution to 1:2 with isopropanol, were set as the optimized ones: (i) a sol-gel liquid pressure of 0.5 bar, (ii) an air pressure of 1.2 bar, (iii) a nozzle coating speed of 3 mm/s and (iv) a nozzle/sample distance of 7 cm. After deposition, the piece was cured by spinning over a platform and exposed to UVA irradiation with a low-pressure mercury lamp for 30 min. Additionally, the application of consecutive layers of the coating was studied, and a thickness of 20 µm could be reached by depositing three consecutive layers while applying the parameters described above.

- Aging study of the sol

Despite the promising properties of the sol-gel layer, there are certain important challenges that need to be overcome before wide industrial applications can be considered. One decisive factor in determining the composition, functionality and integrity of the final coating is the period between solution preparation and coating deposition.

During sol-gel synthesis, the hydrolysis of alkoxides occurs in the presence of water and partially hydrolyzed precursors are formed along with condensation reactions. During the post-synthesis aging, these chemical reactions are not stopped in the system and continue propagating, following the condensation step in which partially hydrolyzed precursors are polymerized, depleting the amount of available silanol groups. The long-term aging effects usually cause destabilization of the sol-gel due to the extended progress of condensation reactions and often even gelation of the formulation before it is applied to a substrate. The intensity of gelation can be quantified by measurement of the viscosity of the solution. Usually, an increase in viscosity can affect the thickness of the layer, and it is possible to reach a critical thickness beyond which the coating losses its integrity and causes partial degradation of the functionality.

**Figure 6:**
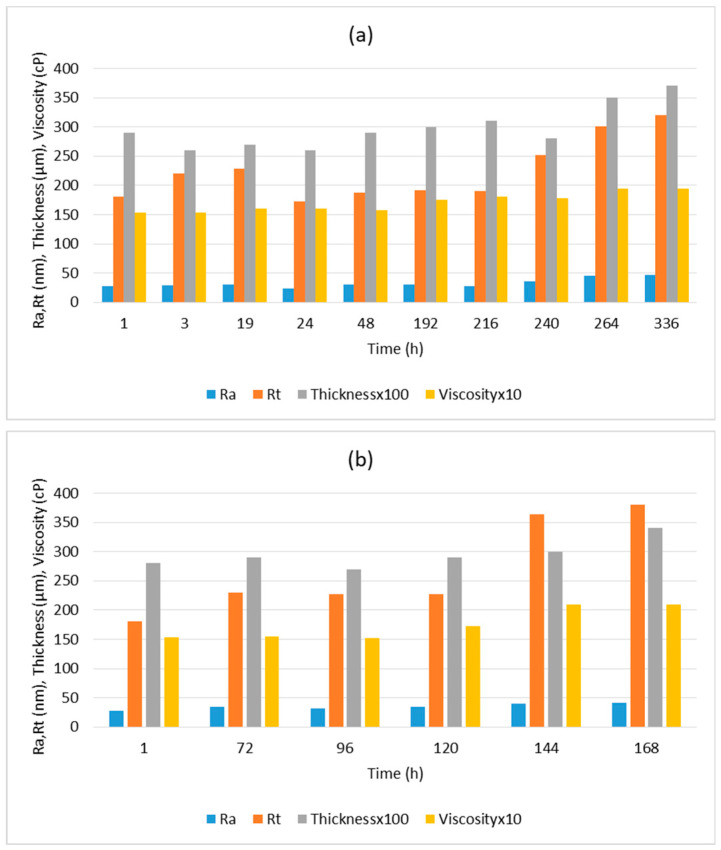
Progress of various parameters over time before and after deposition of the levelling sol-gel coating for determination of the service life: (**a**) in the dark, and (**b**) exposed to light.

In real industrial practice, long storage times are often required. In order to guarantee a long service life of the solution before deposition over large areas, the formulation was scaled up (as specified in Section 2.2.2) and monitored with respect to the viscosity before deposition so that the thickness and roughness parameters could be measured after deposition over the substrate. The data obtained is detailed in Table 4, and graphical monitoring is represented in Figure 6. The solution was stored in the dark during the test to prevent possible curing under visible light.

As can be observed from the analysis of the samples and acquisition of the corresponding data, viscosity and thickness increased suddenly after 240 h of storage. Before this time, a thickness of approximately 3 µm is maintained, with a coating layer that does not present any defect or cracks, thus preserving the desired functionality. Additionally, the Ra and Rt values start to increase at this time, with Ra being >30 nm and Rt being >300 nm (values considered optimal for the levelling effect). The increase in these parameters can be observed in Figure 6, although the increment is quite slow.

The aging stability and corresponding shelf-life of the solution focused on an industrial application could be determined through this study, establishing a period of 240 h (10 days) during which the formulation (stored in the dark) is in optimal conditions to be applied over the substrate. The same study was conducted for the storage under light (as shown in Figure 6b, and a maximum of 120 h (5 days) was determined to guarantee the properties of the coating. Beyond 120 h, viscosity increases, affecting the total roughness, with values of Rt > 300 nm being outside the desired range. Therefore, the functionality of the coating cannot be guaranteed for longer storage periods.

### 3.2. Easy-to-Clean Layer

The main objective of this part of the work was to modify the sol-gel coating formulation by fluorination to obtain hydrophobic surfaces with low surface energy, which have interesting applications in the domestic sector, where self-cleaning ability is often required for many materials.

It is important to take into account that, usually, roughness enhances hydrophobicity depending on the nature of the corresponding flat surface [29]. Therefore, in this work, the smooth topography of the surface plays a negative role in achieving the objective of enhancing hydrophobicity through fluorination.

In order to achieve hydrophobicity functionality of the coating, different fluoroalkyl compounds were studied by adding a certain amount in the appropriate step of the formulation process, just before the organic partner DPHA. These organic groups replace the Hs of the surface OHs of the silica films during the sol-gel process forming a layer on the surface which is non-hydrolyzable and hence hydrophobic.

**Figure 7:**
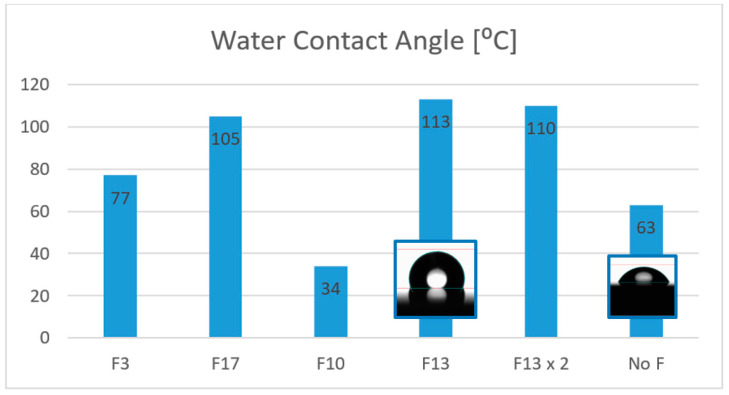
Water contact angles of UV curing sol-gel coatings on AISI 430 by addition of different fluorinated compounds, including photographs of the water droplet of the more representative results.

When a fluoroalcoxysilane is employed, such as 3,3,3-Trifluoropropyl-trimethoxysilane or Triethoxy-1H, 1H, 2H, 2H-heptadecafluorodecylsilane (F3 and F17 respectively in Figure 7), they interconnect with the polysiloxane network, and the contact angle on the surface is increased to 77 and 105°, respectively, making the surface hydrophobic. When employing a different agent with a fluorinated backbone but without silyl ether groups such as 2H, 3H-Decafluoropentane (F10 in Figure 7), the effect obtained is the opposite, as the WCA observed is only 34°, making the surface more hydrophilic than the untreated one (No F in Figure 7). The best result was found when F13 (1H, 1H, 2H, 2H-Perfluorooctyltriethoxysilane) was employed as the hydrophobicity agent, where the WCA could be incremented to 113° and the water droplet adopted a more spherical shape. The molar ratio of F13/MEMO = 1 was optimized to achieve the best result, as it was observed that even doubling the quantity of the chemical led to poorer contact angles (F13x2 in Figure 7).

**Figure 8:**
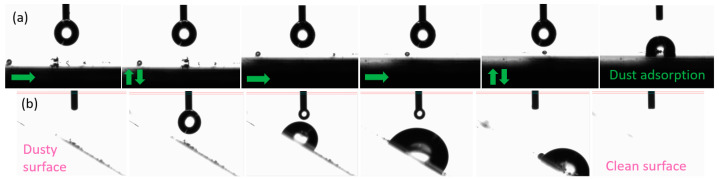
Process of dust absorption test by a water droplet on (**a**) planar hydrophobic surface (arrows indicating the platform moving direction); (**b**) hydrophobic surface at an angle of 40° and consequent sliding of the droplet (when increasing volume) dragging dust.

To test the self-cleaning ability of the coated surface, a more visual experiment was performed and documented by photographs taken with a goniometer. Figure 8 shows the testing process of dust removal by water droplets. The dust was absorbed by the water droplet and collected from the surface, confirming the anti-sticking property of the coating. With a hysteresis angle of 40° and increasing the volume of the droplet [30], it rolls and slides on the inclined hydrophobic surface. It is possible to observe how the water picks up dust particles from the hydrophobic surface, dragging them by sliding down the material.

**Figure 9:**
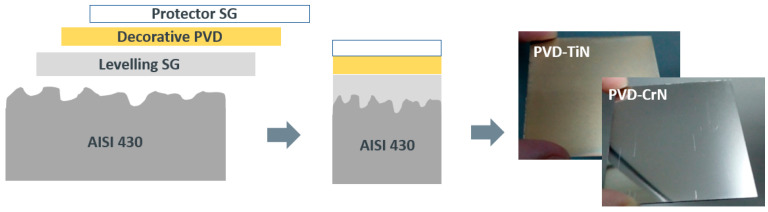
Schematic drawing of the multilayer system and pictures of the gold and silver finish reached under the top coat.

Furthermore, in order to study the application of the coating for domestic pieces resistant to the abrasion of cleaning products, a hydrophobic top coat was deposited onto a multilayer system consisting of an AISI substrate treated with a sol-gel levelling layer and coated by PVD with TiN (gold finish) or CrN (silver finish). The multilayer example is represented in Figure 9.

In order to analyze the effects on the different layers, roughness and WCA were measured after the deposition of each coating, and the results are collected in Table 5 as the average of measurements taken at three points over the surface. Several conclusions can be drawn from the acquired data: (i) the first sol-gel layer works as a levelling coating, reducing the roughness of the AISI 430 substrate to the expected objective (Ra < 30 nm and Rt < 300 nm) and while the surface remains hydrophilic (WCA < 90°); (ii) the silver finish by PVD coating can be deposited over the levelled sol-gel surface through the HIPIMS method, resulting in a significant increase in roughness and, unexpectedly, an intrinsic hydrophobicity (which is transitory and is lost over the time); (iii) the protective sol-gel topcoat leads to a durable hydrophobic layer which also works as levelling layer, reducing Ra and Rt to the same values as the first layer.

- UV irradiation test. Transparency

The coated samples were tested against UV irradiation to determine whether there were color changes and to ensure that there were no alterations of the visual aspect after long periods of time, as well as to assess the durability of hydrophobic/protector functionality. The UV irradiation tests of the films were conducted using a QUV-weathering tester (QUV/Solar Eye Spray). The samples were exposed to UVA irradiation (340 nm) with a power of 0.76 W/m^2^ at 45 °C for 300 h, and the WCA of the samples was measured at different UV irradiation times.

Color data were measured before (sample M in Table 6) and after (samples N-P in Table 6) UV irradiation for different periods of time using a BYK-Gardner spectro-guide sphere color spectrophotometer. The coordinates of the color of samples M, N, O and P, as well as their ΔE values calculated from Equation (1) before and after solar irradiation, are listed in Table 4. ΔE values were calculated using the color data, where L* is the level of light or dark, a* means redness or greenness and b* is the coordinate that indicates yellowness or blueness.

(1) $$\Delta E\;=\sqrt{\left[\Delta L^{2}+\Delta a^{2}+\Delta b^{2}\right]}$$

The ΔE value was measured throughout the time of exposure, and the total ΔE value is expressed in Table 6, which measures the difference in color between sample M and sample P after 300 h under UV radiation. Based on the reviewed bibliography, an ΔE value of less than three indicates that the change in color cannot be perceived by the naked eye [31,32]. Therefore, no distinguishable color difference was observed in the covered AISI 430 surfaces obtained in the scope of this study. The coating studied does not alter the visual aspect of the substrate, and the human eye cannot notice the color change even after long UV exposure times. This makes the coating potentially applicable as a decorative protector film for the domestic sector.

The protector functionality against cleaning products is unaltered after light exposure, as can be verified by the measurement of the water contact angle with a water droplet (which does not change after the aging cycles, as shown in Table 6).

### 3.3. Chemical Analysis of the Coatings

- Thermogravimetric Analysis (TGA)

**Figure 10:**
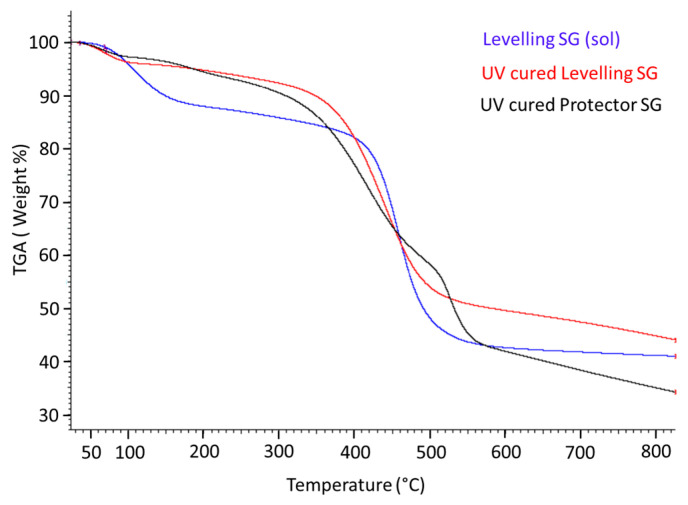
Thermogravimetric analysis (TGA) thermogram for levelling sol-gel coating solution (blue), the UV-cured levelling coating (red) and the UV-cured protector hybrid film (black).

The thermal properties of the hybrid films were evaluated with the TGA analysis to ensure the thermal resistance of the coatings once cured. The thermo-gravimetric analysis was also performed on the uncured sol-gel solution for the levelling formulation to compare differences. When examining the TGA thermogram for the levelling hybrid sol-gel solution (represented in blue color in Figure 10), thermal decomposition follows a two-stage process at 106 °C and 462 °C, with 12% and 47% weight loss, respectively. The TGA analysis for the corresponding cured hybrid film (represented in red in Figure 10) shows a thermal decomposition process following two stages at 66 °C and 442 °C, respectively. The data collected indicated that there was only a 4% loss of weight in the range of 0–140 °C due to the evaporation of residual solvents or moisture trapped in the film, which was eliminated at the beginning of the heating treatment. The 51.7% loss of weight was observed at approximately 442 °C, which is similar to the not sintered coating.

In the case of the protector sol-gel cured coating (represented in black in Figure 10), thermal decomposition follows a four-step pattern in which the loss of weight observed at 57 and 190 °C is a total of 7% loss, assignable to the solvent or volatile compounds evaporation. The maximum loss of weight at 421 °C was 32% loss. Moreover, the TGA thermogram for this film indicates a new decomposition stage around 491 °C (16% loss of weight), which may be associated with the decomposition of sol-gel precursor content F13. The char yields at 800 °C were also collected for all the formulations, showing a 41% residue in the solution levelling formulation, 44% in the cured one, and 34% for the protector sol-gel coating.

This analysis confirms that both formulations are thermally resistant at temperatures up to 350 °C, which is a considerably high resistance for a hybrid sol-gel coating cured under UV light.

- Fourier-transform infrared spectroscopy (FTIR)

**Figure 11:**
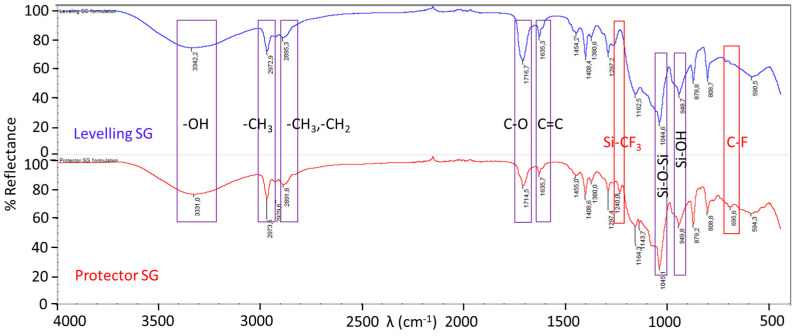
Fourier-transform infrared spectroscopy (FTIR) spectrum for the coating solutions designed for levelling and protector hybrid films.

The FTIR spectrum of the sol-gel coating solutions for the levelling and protector functionalities is represented in Figure 11. Both of them show the typical signals for this kind of organic-inorganic hybrid coating, with the difference being the presence of fluoro-alkylated chain peaks, which are only observed in the protector coating, confirming that the formulation is identical, with the only difference being the addition of this substance. The assignment of the signals is collected in Table 7.

Some of the common absorption bands of a sol-gel coating were found at 1045 cm^−1^, which corresponds to the stretching vibrations of Si-O-Si bonds at 948 cm^−1^, which is assigned to the Si-OH groups formed from hydrolysis of TEOS and the broad band around 3340 cm^−1^ which stands for the asymmetric stretching of various of these OH groups and others coming from moisture, residual solvent, etc. The asymmetric C–H stretching peaks for the methyl groups are located at 2970 cm^−1^ and 2890 cm^−1^, with the latter also being a signal for the C–H stretching peak of the methylene group present in the molecular formula of MEMO and DPHA. The other clearest peak is the stretching vibration peak for the carbonyl group, that was observed at a wavelength of around 1715 cm^−1^ in both formulations. This signal and the one found at 1635 cm^−1^, corresponding to a C=C bond stretching, are assignable to the chemical formula of MEMO. The decreasing of this signal is usually observed in hybrid sol-gel coatings when the curing step takes place, as the C=C of the MEMO reacts with the hexa-functional monomer DPHA, which can polymerize to form a cross-linked network via a photo-sensitive UV-curing process. Nevertheless, this peak does not disappear after curing in our case, presumably due to an excess of MEMO in the formulation.

The signals associated with 13F can be found in the protector sol-gel spectrum at 1240 and 695 cm^−1^ corresponding to the Si-CF_3_ and C-F bond stretching, respectively. Other characteristic peaks of 13F are not in evidence because of overlapping with those of MEMO.

- Energy dispersive X-ray spectroscopy (EDS)

**Figure 12:**
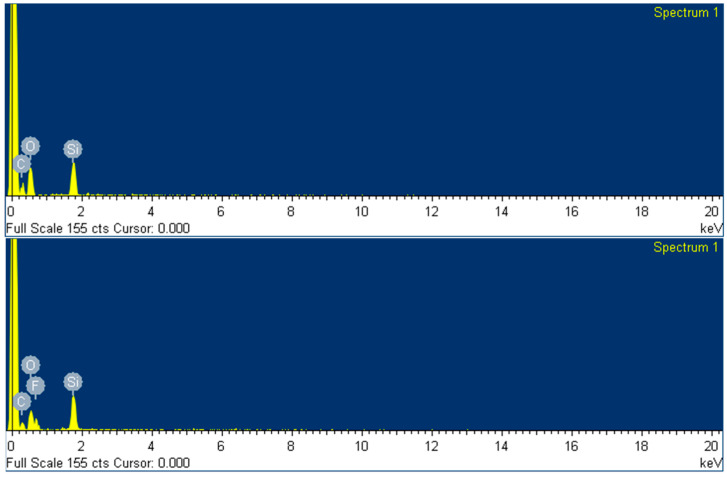
EDS analysis for the levelling sol-gel coating (**top**) and the protector sol-gel coating (**bottom**).

The composition of the surface of the two different coatings was also analyzed by EDS by using the scanning electron microscope (SEM) equipment at 20 kV. The data is presented in Table 8 and represented in Figure 12. The composition was measured as weight and atomic percentage, and only C, O and Si were detected along the area selected, as expected. It was also found that the weight content of fluoride is 20% for the protective coating and inexistent for the levelling formulation, and the quantities of elements C and Si remain unaltered.

## 4. Conclusions

This scalable and low-cost technique for coating stainless steel provides an optimum protective and levelling barrier suitable for many industrial applications. Moreover, a multilayer system was performed to demonstrate the suitability of the method for applying consecutive coatings of different materials deposited by sol-gel and PVD for use in components in the domestic sector.

While hybrid organic-inorganic coatings have been studied before, there are not many examples in the literature showing this dual effect simply by adding an extra additive. Instead of deposition over a polymer, the functionality pursued was studied over AISI 430 as an alternative method for coating in an ultrafast mode without the use of high temperatures that could affect the internal layers of the system. In addition, the intrinsic flexibility of this type of hybrid coating allows for the functionalization of components with different geometries, not restricted to planar samples.

A full characterization of the layers is included, showing good adherence to the substrate, considerable hardness and thermal resistance, as well as accomplishing the functionality pursued in each case. The method is scalable with a simple re-optimization of the parameters and applicable to large areas using the spray coating method. An aging study of the formulation also allows for the guarantee of the solution’s shelf-life before deposition, opening the door to a possible industrialization method of functionalizing stainless steel for many interesting applications.

## Figures and Tables

**Table 1:** AISI 430 stainless steel composition (% wt).

Element	C	S	P	Si	Mn	Cr	Ni	Mo
Content	0.070	0.002	0.023	0.180	0.810	16.21	0.054	≤0.10

**Table 2:** Thickness and roughness of samples covered at different conditions.

Entry	Sample	rpm 1st Layer	rpm 2nd Layer	Coat Thickness 1st Layer (µm)	Total Coat Thickness (µm)	Ra (nm)	Rt (µm)
1	AISI 430	-	-	-	-	81	1.80
2	A	500	-	6.7	6.7	20	0.14
3	B	1000	3000	4.3	7.3	27	0.19
4	C	2000	3000	3.0	5.7	29	0.21
5	D	3000	-	1.7	1.7	23	0.17

**Table 3:** Hardness results of coated samples acquired by nanoindentation.

Sample ^1^	Thickness (µm)	Force (µN)	Hardness (±σ) ^2^ (GPa)	Elastic Modulus (±σ) (GPa)	δ_C_^3^ (nm)
E	1.9 ± 0.1	500	0.332 (±0.006)	5.15 (±0.08)	214
F	1.6 ± 0.2	500	0.392 (±0.016)	5.42 (±0.16)	192
G	1.6 ± 0.2	500	0.332 (±0.007)	5.15 (±0.09)	213
H	1.6 ± 0.1	500	0.306 (±0.006)	4.26 (±0.10)	225
I	1.9 ± 0.1	1000	0.372 (±0.006)	6.73 (±0.13)	305
J	1.6 ± 0.2	1000	0.394 (±0.019)	6.37 (±0.15)	295
K	1.6 ± 0.2	1000	0.370 (±0.009)	6.65 (±0.09)	306
L	1.6 ± 0.1	1000	0.339 (±0.005)	4.77 (±0.06)	322

^1^ A total of 25 indentations were done per sample at the two different forces; ^2^ σ = standard deviation; ^3^ δ_C_ = depth of penetration of the indenter tip.

**Table 4:** Monitoring of different parameters while storage before and after deposition.

Time (h)	Viscosity (cP)	Thickness (µm)	Ra (nm)	Rt (nm)
1	15.4	2.9	28	180
3	15.4	2.6	29	220
19	16.0	2.7	30	229
24	16.0	2.6	24	173
48	15.8	2.9	30	187
192	17.5	3.0	30	192
216	18.0	3.1	28	190
240	17.8	2.8	36	252
264	19.4	3.5	45	301
336	19.4	3.7	47	320

**Table 5:** Monitoring of roughness and WCA for the different coatings of the multilayer system.

Layer	Ra (nm)	Rt (nm)	WCA (°)
AISI 430 untreated	81	1800	88
Levelling SG	28	190	63
PVD Cr-N	39	1191	90–105
Protector SG	17	195	113

**Table 6:** Coordinates of color and ΔE values for samples exposed to UV radiation test.

Sample	Time (h)	WCA (°)	L*	a*	b*	ΔE	ΔE Tot
M	0	113.3	68.26	0.32	2.21	-	-
N	72	113.4	67.70	0.88	2.92	1.06362587	-
O	150	113.6	69.01	0.21	3.24	1.50578883	-
P	300	113.2	67.92	0.33	3.95	1.30636901	1.77293542

**Table 7:** FT-IR wavenumbers and observed signals of the SG coating solutions.

Assignments	Peak Wavenumber (cm^−1^) Levelling SG	Peak Wavenumber (cm^−1^) Protector SG
-OH	3342.2	3331.0
-CH_3_	2972.9	2973.5
-CH_3_, -CH_2_	2895.3	2891.8
-C=O	1716.7	1714.1
-C=C	1635.3	1635.7
-Si-CF_3_	-	1240.0
-Si-O-Si	1044.6	1045.1
-Si-OH	948.7	949.8
-C-F	-	695.6

**Table 8:** Weight % and atomic % elemental composition by EDS of both coatings.

	Weight %					Atomic %		
Element	C	O	F	Si	C	O	F	Si
Levelling SG	24.05	55.04	0.00	20.91	32.36	55.61	0.00	12.03
Protector SG	23.93	35.67	19.58	20.82	33.24	37.20	17.19	12.37

## Data Availability

Not applicable.
